# Nutrition meets nephrology in head and neck cancer: the predictive impact of body composition on the onset of acute kidney disease

**DOI:** 10.3389/fonc.2026.1731918

**Published:** 2026-04-29

**Authors:** Francesco Trevisani, Sara Cardellini, Matteo Floris, Agnese Monti, Alberta Culiersi, Chiara Lucrezia Deantoni, Andrea Pontara, Leone Giordano, Riccardo Caccialanza, Andrea Angioi, Aurora Mirabile

**Affiliations:** 1 Department of Urology and Division of Experimental Oncology, IRCCS San Raffaele Scientific Institute, Milan, Italy; 2 Urological Research Institute, IRCCS San Raffaele Scientific Institute, Milan, Italy; 3 Clinical Nutrition, IRCCS San Raffaele Scientific Institute, Milan, Italy; 4 Department of Nephrology, Dialysis, and Transplantation, ARNAS G. Brotzu, Cagliari, Italy; 5 Department of Otorhinolaryngology, IRCCS San Raffaele Scientific Institute, Milan, Italy; 6 Department of Radiation Oncology, IRCCS San Raffaele Scientific Institute, Milan, Italy; 7 Clinical Nutrition and Dietetics Unit, Department of Oncology, Comprehensive Cancer Center Fondazione IRCCS Policlinico San Matteo, Pavia, Italy

**Keywords:** AKD, AKI (acute kidney injury), chemotherapy - oncology, high-dose cisplatin, LA-HCN

## Abstract

**Background:**

Patients with head and neck cancer undergoing high-dose cisplatin chemotherapy combined with radiotherapy are particularly at risk of developing nephrotoxicity. While Acute Kidney Injury risk is well established, the role of subclinical renal impairment and its potential interplay with nutritional status remains poorly defined. We aim to evaluate how body composition pre-treatment can predict the onset of Acute Kidney Disease.

**Materials and methods:**

We conducted a prospective, monocentric, observational study involving 110 patients with locally advanced HNC treated with concurrent cisplatin (≥200 mg/m²) and radiotherapy. Patients were treated either in a definitive or postoperative adjuvant setting, according to institutional clinical practice. Baseline body composition was assessed by bioelectrical impedance analysis (BIA). Renal function and biochemical data were collected at three time points across 21 days. Variables associated with AKD were identified via univariate analysis (Mann–Whitney U test), followed by multivariate logistic regression for independent predictors.

**Results:**

AKD developed in 20% of patients during treatment. Those who developed AKD exhibited a significantly higher median increase in serum creatinine compared to non-AKD patients (Δcreatinine: 0.48 vs. 0.04 mg/dL, *p* < 0.001). Low body cell mass normalized for height (BCM/kg/m) emerged as a significant independent predictor of AKD (OR = 1.29, *p* = 0.04). A trend toward association was also observed for leukocyte count and platelet levels. Other nutritional indicators (BMI, phase angle, SMI) did not show a significant association. Importantly, all patients completed radiotherapy and reached the target cisplatin dose.

**Conclusions:**

A trend toward an association between low lymphocyte count and reduced muscle mass with AKD development suggests that pre-treatment nutritional and immunological status may influence nephrotoxicity risk. Bioimpedance-derived metrics may provide a non-invasive, reproducible method to identify high-risk patients.

## Introduction

Head and neck cancers (HNCs) represent a diverse group of malignancies originating from the mucosal linings of the upper aerodigestive tract, including the oral cavity, pharynx, and larynx. Globally, over 880,000 new cases and 440,000 deaths were estimated in 2022, making HNCs the seventh most common malignancy worldwide (1). The vast majority (>90%) are squamous cell carcinomas (HNSCC), with a significant proportion diagnosed at a locally advanced stage (LA-SCCHN, stage III–IVB), where cure is still possible but requires multimodal therapy (2, 3).

The standard curative approach for LA-SCCHN is concurrent chemoradiotherapy (CRT), especially for patients who are not surgical candidates or when organ preservation is desirable. The most commonly adopted regimen involves cisplatin at a cumulative dose ≥200 mg/m², typically administered as 100 mg/m² every 3 weeks, in combination with radiotherapy totaling 66–70 Gy delivered with intensity-modulated techniques (IMRT) over approximately 6.5 weeks (4–6). While this strategy improves locoregional control and overall survival, it is associated with high toxicity rates, particularly mucositis, dysphagia, ototoxicity, and nephrotoxicity (7). The challenge is further compounded by the complex anatomy of the head and neck, which renders both tumor progression and treatment-related toxicity highly impactful on nutritional intake, hydration, and functional status.

Cisplatin, a platinum-based chemotherapeutic agent, is effective but nephrotoxic. It induces tubular damage through oxidative stress, inflammation, and mitochondrial dysfunction, leading to acute kidney injury (AKI) in 20–30% of patients. The risk increases in the presence of negative fluid balance, malnutrition, or preexisting renal impairment (8, 9). Electrolyte imbalances, especially hypomagnesemia, are also common and may contribute to further nephrotoxicity and treatment interruptions (10). Importantly, renal dysfunction in this setting may extend beyond overt AKI and evolve into acute kidney disease (AKD), as defined by the 2024 KDIGO criteria, which encompasses abnormalities of kidney structure and/or function lasting ≤3 months, including AKI as a subset. This broader framework captures subacute and persistent renal alterations that may remain clinically under-recognized yet represent a critical vulnerability during CRT, potentially impacting treatment continuity and long-term renal outcomes.

Simultaneously, LA-SCCHN patients are at extremely high risk of developing cancer-associated malnutrition and cachexia due to a combination of tumor-related dysphagia, anorexia, catabolic inflammation, and treatment-induced mucosal injury (11). Pre-treatment malnutrition has been reported in up to 50% of patients, and nearly 80% experience clinically significant weight loss (>5%) during treatment (12). Sarcopenia, defined as loss of skeletal muscle mass and strength, has emerged as a robust predictor of poor outcomes in HNC, being associated with increased CRT-related toxicity, reduced treatment adherence, and decreased overall survival (13, 14) beyond higher rates of recurrence (15). Inflammation-driven alterations in metabolism, appetite regulation, and protein turnover also play a role, and systemic inflammation markers such as the neutrophil-to-lymphocyte ratio (NLR) and C-reactive protein (CRP) have shown prognostic value in this context (16, 17).

Furthermore, the interplay between nutrition and renal function is bidirectional and clinically significant. Malnutrition may reduce renal resilience through loss of lean body mass, hypoalbuminemia, and impaired drug metabolism, while renal impairment may exacerbate anorexia, catabolism, and nutrient losses, creating a vicious cycle that contributes to both early toxicity and long-term morbidity (18). Hypoalbuminemia, in particular, is an independent predictor of both nephrotoxicity and mortality, underscoring the need for early nutritional and biochemical assessment (19).

To mitigate these risks, guidelines increasingly recommend a multidisciplinary approach to patient care, including early nutritional assessment, individualized nutrition therapy, renal monitoring, and supportive interventions such as prophylactic gastrostomy placement, electrolyte supplementation, and modified fluid replacement protocols (20–22). Despite these advances, real-world adherence to such comprehensive strategies remains variable, and evidence on how nutritional and renal trajectories evolve across the treatment continuum is still limited.

In our previous work (23), we performed a pilot observational study on a discovery cohort of advanced head and neck cancer patients undergoing cisplatin- based chemoradiotherapy, showing the longitudinal relationship between renal function decline and nutritional management. However, due to the limited number of observations, no predictive nutritional markers of acute kidney damage onset were discovered.

Therefore, the present prospective observational study, based on a cohort of 110 patients undergoing standard cisplatin-based CRT supported by structured nutritional assessments and renal monitoring, aimed to fulfill this clinical gap by highlighting innovative clinical variables able to stratify, at baseline, the high-risk patient group in terms of progressive renal dysfunction.

## Materials and methods

A consecutive cohort of 110 patients was enrolled between May 2021 and April 2025 at IRCCS San Raffaele Hospital in Milan. Clinical and biochemical data were collected at 3 time points (from 0 to 21 days); anthropometric measures and body composition were evaluated at the beginning of the treatment. This study included adults aged ≥ 18 years with LA-HCN deemed medically fit for a treatment plan involving either primary or adjuvant radiotherapy with concurrent chemotherapy (high-dose cisplatin). Treatment intent (definitive vs postoperative adjuvant) was recorded at baseline and included as a categorical descriptor in baseline characteristics. Women known to be pregnant or planning to become pregnant during the trial period, as well as patients requiring total parenteral nutrition, were excluded from the study.

The study received the approval of the Institutional Ethical Committee (San Raffaele Hospital, Milan, approval date 08/06/2022), and all patients included in this study signed an informed consent form. All the experimental procedures involving human biological material complied with the approved guidelines and were conducted according to good clinical practice.

### Chemoradiotherapy

All patients were treated with helical TomoTherapy^®^ (HT- Accuray, Maddison, WI, USA). All patients included in the analysis were treated with a standard radiotherapy fractionation schedule; accelerated radiotherapy was not administered in this cohort. In a radical setting, an 18Fluorodeoxyglucose computed tomography (CT) positron emission tomography (PET) was performed to identify the biological target volume (BTV). A hypofractionated schedule with a simultaneous integrated boost (54 Gy in 30 fractions on bilateral neck nodes and 66 Gy on tumor and high-risk/PET-positive nodes) was used in these patients. These patients received primary concomitant Cisplatin during standard fractionated RT (or on day 1 and 22 during accelerated RT) (24, 25). In the postoperative setting, RT was prescribed according to histological examination (54 Gy in 30 fractions on low-risk volumes and 61.5–64 Gy in 30 fractions on high-risk volumes). These patients underwent Cisplatin 100 mg/m^2^ on days 1, 22, and 43 during RT or Cisplatin 50 mg flat dose weekly according to the patient’s performance status. The cisplatin administration schedule (100 mg/m² every 3 weeks or weekly cisplatin) was selected based on individual clinical characteristics, including patient frailty and non-nephrological comorbidities, in accordance with institutional practice, and was not driven by treatment intent. In any case, patients received at least 200 mg/m^2^ of cisplatin (26).

### Nutritional assessment

Each patient underwent a nutritional evaluation before the treatment. Total daily energy requirements were calculated by multiplying the estimated resting energy expenditure (using the Harris‐Benedict equations) by a correcting factor of 1.5. For each patient, body weight and height were collected as well as the weight lost 3-6months before the visit; as described by the World Health Organization (27), BMI was calculated by dividing body weight (kg) by height squared (m2). BIA 101 BIVA bioelectric impedance device (Akern^®^) was used to obtain the following parameters: Standardized Phase Angle (SPA), Body Cell Mass Index (BCMI), Free Fat Mass (FFM), Fat Mass (FM), Total Body Water (TBW), Extracellular Water (ECW), Skeletal Muscle Index (SMI) (28). Body Cell Mass normalized for height (BCM/kg/m) was calculated to provide a size-adjusted estimate of metabolically active tissue. BIA measurements were performed under standardized conditions and are reproducible according to manufacturer and published validation studies.

### Nephrological assessment

The patient cohort was stratified according to renal function, defined by the glomerular filtration rate (GFR) following the kidney disease: Improving Global Outcomes (KDIGO) guidelines 2012. Estimated GFR (eGFR) was calculated using the CKD-EPI 2009 equation, based on serum creatinine, age, sex, and ethnicity. Patients were categorized into the following CKD classes: G1 (≥90 mL/min/1.73 m²), G2 (60–89 mL/min/1.73 m²), G3 (30–59 mL/min/1.73 m²), and G4 (15–29 mL/min/1.73 m²). In addition, the onset of Acute Kidney Disease (AKD) was assessed. Acute kidney disease (AKD), according to KDIGO 2024 criteria, is defined as abnormalities of kidney structure and/or function with a duration of ≤3 months, including acute kidney injury (AKI) as a subset, and characterized by decreased glomerular filtration rate and/or markers of kidney damage. For analytical purposes, patients were classified as AKD = 1 if AKD occurred at any time during treatment, and AKD = 0 if AKD was not observed. CKD stratification and the incidence of AKD were evaluated at each time interval following the chemotherapy cycles.

### Statistical analysis

Statistical analyses were performed with JASP version 0.18 with two-sided tests and a significance threshold of P < 0.05. Continuous variables were summarized as median with interquartile range since most of the variables ‘ distributional assumptions were evaluated by the Shapiro–Wilk test and inspection of Q–Q plots. Variables exhibiting significant skewness underwent natural‐log or Box-Cox transformation before inferential testing. Between‐group comparisons of normally distributed continuous measures used Student’s two‐sample t test, whereas the Wilcoxon rank‐sum test was applied to nonparametric data. Categorical variables were compared by χ² testing or, when expected counts were low (<5), by Fisher’s exact test.

Multivariate analysis was carried out with a logistic‐regression model whose estimates are expressed as odds ratios. The primary outcome, AKD, was defined as per KDIGO 2024 criteria.

## Results

Descriptive statistics of baseline variables (demographic, laboratory, and body‐composition measures) are shown in **Table 1**.

**Table 1:** Descriptive statistics of baseline continuous variables (expressed as median, range, and interquartile range) for demographic, laboratory, and body‐composition measures in our study cohort (n = 110).

Variable	Valid	Median	Min	Max	25%	75%
Age (years)	107	61.00	26.00	80.00	52.00	69.50
Weight (kg)	110	72.05	40.90	113.50	60.63	80.40
Height (m)	110	1.68	1.48	1.92	1.62	1.74
BMI (kg/m²)	110	24.65	16.10	38.30	22.30	28.10
Creatinine 1 (mg/dL)	102	0.89	0.42	1.39	0.73	1.00
Creatinine 2 (mg/dL)	102	0.88	0.46	2.24	0.74	1.07
Creatinine 3 (mg/dL)	99	0.97	0.49	2.03	0.74	1.07
eGFR 1 (mL/min/1.73 m²)	102	89.03	42.22	136.91	79.36	100.31
eGFR 2 (mL/min/1.73 m²)	102	84.58	21.82	140.71	69.90	98.72
eGFR 3 (mL/min/1.73 m²)	99	78.62	34.60	129.33	63.07	97.65
Leukocytes (10^9^/L)	104	7.85	2.80	18.30	5.77	9.45
Hemoglobin (g/dL)	104	13.95	8.50	16.90	12.55	14.80
Platelets (10^9^/L)	104	256.00	117.00	681.00	209.50	327.25
Neutrophils (10^9^/L)	102	4.98	0.60	12.40	3.40	6.36
Lymphocytes (10^9^/L)	102	1.80	0.11	17.40	1.40	2.30
Glucose (mg/dL)	82	95.50	64.00	155.00	90.00	109.00
AST (U/L)	98	21.05	10.00	107.00	18.00	25.00
ALT (U/L)	97	22.00	8.00	72.00	16.00	30.00
GGT (U/L)	32	25.00	11.00	104.00	19.00	38.25
ALP (U/L)	73	75.00	37.00	225.00	67.00	90.00
LDH (U/L)	71	186.00	74.00	390.00	164.50	221.00
Urea (mg/dL)	44	34.00	17.00	67.00	26.50	42.50
Total Bilirubin (mg/dL)	78	0.53	0.22	1.85	0.40	0.69
Total Protein (g/L)	75	69.70	6.70	84.50	66.55	72.00
Iron (µg/dL)	69	81.00	16.00	176.00	54.00	100.00
Prealbumin (g/L)	64	0.25	0.11	0.37	0.21	0.28
Albumin (g/L)	70	41.80	31.60	49.00	37.45	44.00
Weight Loss (kg, 3–6 months)	110	4.00	0.00	30.00	0.00	7.00
Phase Angle (°)	110	5.50	3.60	7.30	4.90	6.10
Phase Angle Difference (°)	110	-0.80	-3.00	1.10	-1.28	-0.40
Body Cell Mass Index (BCMI)	110	10.10	5.60	15.60	8.45	11.78
Basal Metabolic Rate (BMR, kcal)	110	1602.55	1183.70	2073.70	1426.95	1754.45
Total Energy Expenditure (TEE, kcal)	109	2428.40	1775.50	3209.80	2142.40	2634.70
Body Cell Mass (BCM, kg)	110	29.70	15.00	45.60	23.33	34.63
BCM per meter (kg/m)	74	17.25	8.90	26.80	15.20	20.48
Fat-Free Mass (FFM, kg)	110	58.05	33.40	82.50	48.10	65.07
Fat-Free Mass (%)	110	80.35	52.70	95.90	74.63	85.17
Δ Fat-Free Mass (%)	110	4.35	-19.60	31.00	0.30	9.10
Fat Mass (kg)	110	14.30	1.90	51.50	10.05	18.10
Fat Mass (%)	110	19.65	4.10	47.30	14.82	25.37
Δ Fat Mass (%)	110	-4.25	-31.00	19.60	-8.47	-0.30
Total Body Water (L)	110	42.90	24.30	61.00	35.15	49.25
Δ TBW (% of weight)	110	1.60	-12.70	67.80	-1.48	4.68
Extracellular Water (ECW, L)	110	20.10	12.50	31.70	17.72	23.00
Δ ECW (% of TBW)	109	2.00	-4.70	14.30	-0.50	5.20
Skeletal Muscle Mass (SM, kg)	102	29.30	12.60	42.30	23.13	33.25
Skeletal Muscle Index (SMI, kg/m²)	102	10.10	5.70	14.60	8.53	11.20
Appendicular SMM (ASMM, kg)	102	21.65	11.20	33.00	18.25	25.37
Fat-Free Mass Index (FFMI, kg/m²)	102	20.10	14.00	27.30	18.00	22.10
Fat Mass Index (FMI, kg/m²)	102	4.95	0.80	17.40	3.50	6.65
Standardized Phase Angle (SPA, °)	102	0.19	-2.26	2.76	-0.62	0.73

BMI, Body Mass Index; eGFR, estimated Glomerular Filtration Rate; AST, Aspartate Aminotransferase; ALT, Alanine Aminotransferase; GGT, Gamma-Glutamyl Transferase; ALP, Alkaline Phosphatase; LDH, Lactate Dehydrogenase; PhA, Phase Angle; BCM, Body Cell Mass; FFM, Fat-Free Mass; FM, Fat Mass; TBW, Total Body Water; ECW, Extracellular Water; SMM, Skeletal Muscle Mass; FFMI, Fat-Free Mass Index; FMI, Fat Mass Index.

Creatinine 1/2/3 and eGFR 1/2/3 refer to serum creatinine and corresponding eGFR values measured at standardized pre-treatment time points. For patients receiving cisplatin every 3 weeks, measurements were obtained prior to cycles 1, 2, and 3. For patients receiving weekly cisplatin, measurements were aligned at approximately three-week intervals (prior to the 1st, 3rd, and 6th weekly cycles) to ensure temporal comparability across regimens.

Distribution of categorical variables stratified by AKD (**Table 2**) demonstrated that obesity (BMI > 30 kg/m2) appears to have no association with the onset of AKD during treatment. AKD is defined as the occurrence of AKD at any time during treatment (yes/no).

**Table 2:** Distribution of categorical variables stratified by AKD, with counts, percentages, and χ² test for independence.

Characteristic	AKD = 0, n (%)	AKD = 1, n (%)	Total, n (%)	p value¹
Sex				0.15
Female	25 (31.6)	4 (16.7)	29 (27.6)	
Male	54 (68.4)	20 (83.3)	74 (72.4)	
CKD Stage (eGFR)				0.31
G1 (≥ 90)	39 (49.4)	11 (45.8)	50 (47.1)	
G2 (60 – 89)	33 (41.8)	9 (37.5)	42 (39.6)	
G3a (45 – 59)	4 (5.1)	4 (16.7)	8 (7.5)	
G3b (30 – 44)	1 (1.3)	0 (0)	1 (0.9)	
BMI Classification (kg/m^2^)				0.12
Underweight (< 18.5)	3 (3.8)	1 (4.2)	4 (3.8)	
Normal (18.5 – 24.9)	40 (50.6)	10 (41.7)	50 (47.1)	
Overweight (25 – 29.9)	27 (34.2)	7 (29.2)	34 (32.0)	
Obesity I (30 – 34.9)	5 (6.3)	1 (4.2)	6 (5.7)	
Obesity II (35 – 39.9)	1 (1.3)	0 (0)	1 (0.9)	
Obesity III (≥ 40)	1 (1.3)	0 (0)	1 (0.9)	

¹χ² test for independence.

Other clinical parameters (regarding liver function, glycemia, and iron, for example) and data obtained from BIA (for example, SPA, FFMI, SMI, and ASMM) didn’t show a significant impact on the onset of AKD during treatment.

Considering the univariate comparison (**Table 3**) and Multivariate logistic‐regression models (**Table 4**), BCM (kg/m) seems the only variable with a strong correlation with AKD, but a trend is evident even considering leukocytes (p=0.03) and platelets (p=0.05).

**Table 3:** Univariate comparison of continuous variables with AKD (yes/no) using Student’s t–test or Mann–Whitney U test, as appropriate.

Variable	Student’s t	Mann-Whitney	p
Age (years)	-1.07		0.29
BMI (kg/m²)	-1.07		0.29
eGFR 1 (mL/min/1.73 m²)	0.74		0.46
**eGFR 2 (mL/min/1.73 m²)**	**3.38**		**<0.001**
**eGFR 3 (mL/min/1.73 m²)**	**7.22**		**<0.001**
**Leukocytes (10^9^/L)**	**-2.21**		**0.03**
Hemoglobin (g/dL)	-0.14		0.89
**Platelets (10^9^/L)**		**682.5**	**0.05**
Neutrophils (10^9^/L) *	-1.55		0.23
Lymphocytes (10^9^/L)		740.0	0.19
Glucose (mg/dL)		461.0	0.49
AST (U/L)		952.5	0.39
ALT (U/L)		886.5	0.69
GGT (U/L)		42.5	0.20
ALP (U/L)		406.0	0.57
LDH (U/L)		328.5	0.23
Urea (mg/dL)	-0.17		0.87
Total Bilirubin (mg/dL)		548.0	0.45
Total Protein (g/L)		532.5	0.19
Iron (µg/dL)	0.91		0.37
Prealbumin (g/L)	-0.05		0.96
Albumin (g/L)	1.62		0.11
Weight Loss (kg, 3–6 months)		1000.0	0.68
Phase Angle (°)	-0.02		0.98
Phase Angle Difference (°)*	-0.19		0.85
Body Cell Mass Index (BCMI)	-1.12		0.27
Basal Metabolic Rate (BMR, kcal)	-1.24		0.22
Total Energy Expenditure (TEE, kcal)	-1.10		0.28
Body Cell Mass (BCM, kg)	-1.24		0.22
**BCM per meter (kg/m)**	**-2.49**		**0.02**
Fat-Free Mass (FFM, kg)	-1.37		0.17
Fat-Free Mass (%)	0.02		0.99
Δ Fat-Free Mass (%)*	0.74		0.46
Fat Mass (kg)*	-0.69		0.49
Fat Mass (%)	-0.02		0.99
Δ Fat Mass (%)*	0.96		0.35
Total Body Water (L)	-1.15		0.25
**Δ TBW (% of weight)**		**1196.5**	**0.05**
Extracellular Water (ECW, L)	-0.93		0.35
Δ ECW (% of TBW) *	**-0.65**		**0.52**
Skeletal Muscle Mass (SM, kg)	-0.98		0.33
Skeletal Muscle Index (SMI, kg/m²)	-0.85		0.40
Appendicular SMM (ASMM, kg)	-0.94		0.35
Fat-Free Mass Index (FFMI, kg/m²)	-1.19		0.24
Fat Mass Index (FMI, kg/m²) *	-0.49		0.63
Small Phase Angle (SPA, °)	0.99		0.32

*Normalized with Box-Cox correction.

Normality assessed (Shapiro-Wilk test) and Box–Cox correction applied where indicated (*).

Bold values indicate statistically significant results (p < 0.05).

**Table 4:** Multivariate logistic‐regression models predicting AKD, showing model intercepts, variable estimates, standard errors, odds ratios (OR), z‐scores, Wald statistics, degrees of freedom, and p values.

Model		Estimate	Standard error	OR	z	Wald	df	p
M_0_	(Intercept)	-1.50	0.31	0.22	-4.87	23.75	1	1.10×10^-6^
M_1_	(Intercept)	-8.83	3.01	1.47×10^-4^	-2.93	8.60	1	3.37×10^-3^
	Leucocytes (10^9/L)	0.17	0.16	1.19	1.06	1.12	1	0.29
	Platelet (10^9/L)	0.00	0.00	1.00	0.80	0.64	1	0.43
	**BCM (kg/m)**	**0.26**	**0.12**	**1.29**	**2.08**	**4.31**	**1**	**0.04**
	TBW (% weight) difference	-0.09	0.08	0.91	-1.10	1.22	1	0.27
	ECW (% TBW) difference	0.12	0.12	1.13	0.95	0.91	1	0.34

Bold values indicate statistically significant results (p < 0.05).

Other clinical parameters (regarding liver function, glycemia, and iron, for example) and data obtained from BIA (for example, SPA, FFMI, SMI, and ASMM) didn’t show a significant impact on the onset of AKD during treatment.

The majority of patients (95.65%) received the same chemotherapy dose of cisplatin 100mg/m2 evergreen three weeks. Therefore, the weekly schedule, administered in a non significant number of patients and with a cumulative cisplatin dose equal to or greater than 200mg/m², does not represent a confounding factor in the development of nephrotoxicity (**Tables 5**, **6**).

**Table 5:** Distribution of primary tumor sites (N = 110).

Tumor site	N (%)
Oropharynx	40 (36,36 %)
Nasopharynx	17 (15,45 %)
Larynx	16 (14,54 %)
Oral cavity	12 (10,91 %)
Hypopharynx	7 (6,36 %)
NCUP	3 (2,73 %)
Salivary glands	2 (1,82 %)
Maxillary sinus	2 (1,82 %)
Parotid gland	2 (1,82 %)
Nasal cavity SCC	1 (0,91 %)
Papillary thyroid carcinoma	1 (0,91 %)
Salivary gland tumor	1 (0,91 %)
margine linguale sinistro margine linguale sinistro Nose	1 (0,91 %)
Retromolar trigone	1 (0,91 %)
External auditory canal	1 (0,91 %)
Left base of tongue	1 (0,91 %)
Tongue	1 (0,91 %)
Left lateral tongue	1 (0,91 %)

**Table 6:** Association between baseline comorbidities and body cell mass index (BCMI).

Variable	Group	N	BCMI, mean ± SD	Mean difference (95% CI)	P Value
Diabetes	Yes	4	10.45 ± 1.95	0.26 (−1.95 to 2.47)	0.82
	No	106	10.19 ± 2.20		
Untreated Hyperglycemia	Yes	14	11.83 ± 1.55	1.87 (0.68 to 3.06)	0.002
	No	96	9.96 ± 2.16		
Hypertension	Yes	39	10.51 ± 2.12	0.48 (−0.38 to 1.34)	0.27
	No	71	10.03 ± 2.21		

*Untreated hyperglycemia was defined as elevated baseline fasting glucose in patients without a previous diagnosis of diabetes and not receiving hypoglycemic therapy.

BCMI did not differ significantly between patients with and without diabetes (10.45 ± 1.95 vs 10.19 ± 2.20; mean difference 0.26, 95% CI −1.95 to 2.47; p = 0.816). Similarly, no significant difference in BCMI was observed between hypertensive and non-hypertensive patients (10.51 ± 2.12 vs 10.03 ± 2.21; mean difference 0.48, 95% CI −0.38 to 1.34; p = 0.269).

In contrast, patients with untreated hyperglycemia had significantly higher BCMI compared with those without untreated hyperglycemia (11.83 ± 1.55 vs 9.96 ± 2.16; mean difference 1.87, 95% CI 0.68 to 3.06; p = 0.002).

## Discussion

Our work focused on renal function decline during high-dose cisplatin-based chemotherapy in patients with locally advanced squamous cell carcinoma of the head and neck (LA-SCCHN), focusing primarily on acute kidney disease (AKD) and shifts in chronic kidney disease (CKD) stages, while incorporating key nutritional and body composition variables to identify novel risk modifiers. AKD, as outlined by KDIGO guidelines, defines a decline in renal function that occurs up to 90 days following a renal insult (such as a chemotherapy cycle), encompassing progressive and often subclinical dysfunction that escapes traditional acute kidney injury criteria. This endpoint is becoming relevant in oncology, where serum creatinine is frequently measured at baseline and before each chemotherapy cycle, but seldom within the 48–72-hour window required to diagnose AKI.

This limitation is particularly relevant in populations with altered body composition, where creatinine-based estimates may be biased.

Therefore, AKD may offer a more sensitive framework to detect cumulative subacute renal impairment that may influence toxicity and long-term kidney health (29, 30).

In our study, AKD developed in 10.0% of patients by the end of the first chemotherapy cycle, 14.0% by the second, and 20.0% cumulatively after three cycles. These incidence rates are lower compared to those reported in prior cisplatin-treated cohorts, where AKI or AKD rates ranged between 25% and 35%, especially among older individuals, those with baseline renal impairment, or comorbidities like diabetes or hypertension, and in settings with less stringent hydration protocols (31–34).

A key factor leading to the reduction of AKD incidence among the study population appears to be our institution’s rigorously standardized fluid replacement and supportive care regimen, which may explain why renal outcomes here are improved in respect to previous studies. All enrolled patients received ≥1000 mL of isotonic saline pre-cisplatin infusion and ≥1000 mL within six hours after administration, with intravenous magnesium and potassium supplementation standardized across cycles. In select cases, low-dose loop diuretics were administered to enhance fluid turnover and prevent cisplatin accumulation in the renal tubules. This protocol is consistent with findings from meta-analyses demonstrating a reduction of cisplatin nephrotoxicity by approximately 40–60% (35–37) and is also supported by recent evidence from a large multicenter cohort study, about the protective role of prophylactic IV magnesium on cisplatin-related AKI, with an adjusted odds ratio of 0.80 (95% CI, 0.66–0.97) (38), highlighting the importance of micronutrient supplementation as a key part of cisplatin nephroprotection. Additionally, new supportive therapies, such as amifostine, N-acetylcysteine, and high-dose mannitol, have shown mixed results in reducing kidney damage.

Beyond AKD, our analysis also investigated the diverse incidence of CKD among the population before and after treatment. At baseline, 22.0% of patients were in stage G1 (eGFR ≥ 90 mL/min/1.73 m²), 62.0% were G2 (60–89), 10.0% were G3a (45–59), and 6.0% were G3b (30–44). After three treatment cycles, distribution shifted significantly, with only 4.0% of patients in G1, 68.0% in G2, and increases to 20.0% and 8.0% in G3a and G3b, respectively. Median eGFR declined consistently across cycles, from 89.03 at baseline to 84.58 after cycle two and 78.62 after cycle three, representing a median loss of 10.41 mL/min in total, with cycle-specific declines of 4.45 and 5.96 mL/min. Though these changes were not clinically relevant, they represented a consistent, measurable decline in renal function without progression to CKD stage G5 (< 15 mL/min/1.73 m²) or the need for renal replacement therapy, among the 7.5% of patients who began treatment in G3. These findings challenge current clinical guidelines, often excluding individuals with baseline eGFR below 60 mL/min from high-dose cisplatin therapy. Importantly, patients with baseline eGFR <60 mL/min/1.73 m² were able to complete treatment, partly reflecting the inclusion of weekly cisplatin schedules. However, this supports the concept that renal tolerance is multifactorial and not exclusively determined by baseline eGFR. Therefore, the authors confirm the emerging literature’s evidence proposing an individualized eligibility criteria panel based on renal reserve and physiological markers rather than solely eGFR thresholds and subsequent CKD classes (39, 40).

The most influential predictor of AKD in our multivariate analysis was low body cell mass normalized for height (BCM/kg/m), with an odds ratio of 1.29 (p = 0.04). BCM should therefore be interpreted as a possible risk stratification biomarker rather than a causal determinant of AKD, as systemic inflammation may contribute to both BCM reduction and renal vulnerability.

BCM, which reflects the volume of metabolically active lean tissue, is well known to influence pharmacokinetics, protein reservoir, and tissue regenerative capability. While prior oncology research has highlighted the association between low lean mass and increased chemotherapy toxicity or mortality (41–43) the relationship between BCM and renal outcomes has been largely unexplored. In this study, reduced BCM likely contributed to enhanced nephrotoxicity by yielding higher effective cisplatin exposure per unit of renal tissue due to reduced distribution volume, alongside diminished antioxidant and repair capacity secondary to protein depletion (36, 44, 45). In contrast, common body composition metrics such as BMI, phase angle, fat-free mass index, total body water, and extracellular water did not significantly predict AKD, reinforcing the superior physiological and prognostic relevance that BCM could represent in this context. These results echo studies showing that lean mass measurements outperform body surface area in forecasting chemotherapy pharmacodynamics and toxicity (44, 46). Given the central role of BCM, bioelectrical impedance analysis offers a practical, non-invasive approach to identify patients at increased risk for nephrotoxicity and to guide personalized intervention.

It is not possible to draw firm causal conclusions given that the study is observational. However, these promising findings are hypothesis-generating and suggest a potential mechanism that warrants more extensive investigation in future studies.

Our analysis further underscores the importance of nutritional health and sarcopenia in treatment outcomes. BIA-derived parameters, phase angle, BCMI, FFMI, and BCM, are well-established markers of malnutrition and functional decline in head and neck cancer (47), while cisplatin nephrotoxicity is recognized to proceed through a complex combination of tubular uptake, mitochondrial dysfunction, inflammation, and oxidative damage (48). Low serum albumin and prealbumin at baseline have been associated with greater toxicity and reduced chemotherapy completion in high-dose cisplatin regimens (49), reinforcing the need for sustained nutritional intervention to prevent sarcopenia and cachexia. Sarcopenia continues to be widely recognized for its negative impact on prognosis, therapy response, mobility, and overall quality of life among head and neck cancer patients (50).

Even within a cohort receiving standard high-protein diets per ESPEN guidelines (26), we observed only minor changes in body weight and anthropometric indices during treatment. Nonetheless, the modest, yet quantifiable, decline in eGFR did not correlate with overt nutritional deterioration (49). Further supporting the prognostic utility of BCM, the VALOR^®^ study demonstrated that BCM greater than 17 kg/m² is an independent predictor of survival in HNC patients undergoing radiotherapy (47), suggesting that BCM serves as both a renal and oncologic prognostic indicator.

While the long-term renal consequences of cisplatin-induced AKI remain a topic of debate, existing evidence indicates that even transient AKI episodes may predispose patients to progressive renal decline (51). Achievement of cumulative cisplatin dosages ≥200 mg/m² is correlated with improved survival outcomes in LA-SCCHN treated with chemoradiation, though baseline renal function remains a critical determinant of dose completion. In the present study, obesity (BMI >30 kg/m²) was not associated with increased AKD risk, implying that nephrotoxicity in our cohort was influenced more by chemotherapy exposure and physiological reserve than by baseline adiposity.

From a pathophysiology point of view, the gradual decline in eGFR during high-dose cisplatin regimen aligns more closely with cumulative subclinical nephron injury, driven by intracellular oxidative and mitochondrial stress, rather than intermittent acute tubular necrosis. This interpretation is supported by preclinical models demonstrating mitochondrial tubular damage in the absence of overt AKI (52), and human histopathologic data confirming early tubular dysfunction after multiple cisplatin doses (53). Our serial renal monitoring, which provided high-resolution trajectories of renal function over three time points, allowed us to capture these patterns and to advocate for the use of AKD as a relevant endpoint in onco-nephrology studies, while identifying BCM as a pragmatic predictive biomarker (54, 55).

### Strengths and limitations

This study has several notable strengths. It represents one of the few prospective investigations assessing the relationship between pre-treatment body composition and the development of acute kidney disease (AKD) in patients with head and neck cancer undergoing high-dose cisplatin-based therapy. The systematic and longitudinal collection of biochemical, nutritional, and renal parameters, combined with bioelectrical impedance analysis (BIA), enabled a comprehensive assessment of dynamic risk factors. In addition, all patients were managed according to a standardized hydration and electrolyte protocol, including routine intravenous magnesium supplementation, thereby strengthening internal validity and ensuring consistency with current best clinical practices.

However, several limitations should be acknowledged. Although clinically favorable, the relatively low incidence of AKD in our cohort may have reduced the statistical power to identify additional significant associations. Furthermore, this was a single-center study, which may limit the generalizability of the findings to other settings with different patient characteristics or supportive care strategies. While the prospective design is a strength, the observational nature of the study precludes causal inference, and residual confounding cannot be entirely excluded. Moreover, given the limited number of AKD events, the inclusion of a larger number of baseline covariates in the multivariable model would have increased the risk of overfitting; therefore, the analysis was intentionally restricted to a limited set of predictors, and the results should be interpreted with caution.

Finally, although BIA is a practical, non-invasive, and reproducible method, it remains an indirect surrogate of muscle mass and renal reserve. Future research should aim to validate BIA-derived parameters against gold-standard imaging techniques, such as CT-derived skeletal muscle indices, and to investigate whether integrating body composition profiling into nephrotoxicity risk models can enhance predictive accuracy and support the development of personalized supportive strategies in oncologic patients.

## Conclusion

Our findings suggest that the nephrotoxic effects of cisplatin may be attenuated through standardized supportive care and individualized risk assessment incorporating body cell mass (BCM). In this cohort, selected patients with moderate CKD were able to complete treatment without severe renal deterioration; however, these findings should be interpreted with caution in light of the limited sample size and number of events, and cannot be extended to broader clinical settings. Although BCM was associated with AKD risk, its role as a clinical decision-making tool requires further validation. Prehabilitation strategies aimed at preserving BCM, such as nutritional support, resistance exercise, and anabolic interventions, may improve treatment tolerance, but their effect on renal outcomes remains uncertain. Further prospective studies are needed to evaluate whether BCM-informed strategies or dose adjustments can reduce AKD incidence while preserving renal function and treatment efficacy in higher-risk populations.

## Data Availability

The raw data supporting the conclusions of this article will be made available by the authors, without undue reservation.
